# Correction: Effect of the HXBM408 bacteria on rat intestinal bacterial diversity and the metabolism of soybean isoflavones

**DOI:** 10.1371/journal.pone.0303548

**Published:** 2024-05-07

**Authors:** Xie Jinglong, Li Xiaobin, Zhao Fang, Wang Chenchen, Yang Kailun

The authors are listed out of order. Please view the correct author order, affiliations, and citation here:

Xie Jinglong^1☯^, Li Xiaobin^1☯^, Zhao Fang^1‡^, Wang Chenchen^1‡^, Yang Kailun^1^*

^☯^ These authors contributed equally to this work.

^‡^ ZF and WC also contributed equally to this work.

**1** Xinjiang Key Laboratory of Meat & Milk Production Herbivore Nutrition, Xinjiang Agricultural University, Urumqi, China

Jinglong X, Xiaobin L, Fang Z, Chenchen W, Kailun Y (2021) Effect of the HXBM408 bacteria on rat intestinal bacterial diversity and the metabolism of soybean isoflavones. PLoS ONE 16(7): e0253728. https://doi.org/10.1371/journal.pone.0253728
