# Supplementary material for: Effect of the HXBM408 bacteria on rat intestinal bacterial diversity and the metabolism of soybean isoflavones
Source: PLoS One. 2021 Jul 15;16(7):e0253728. doi: 10.1371/journal.pone.0253728 (PMC8282076; doi:10.1371/journal.pone.0253728)
Supplement: Attachment — Submitted filename: Response to reviewers.doc [file pone.0253728.s001.doc]

1. There are several abbreviations in the manuscript that have no full name annotation, like “OTU” in page 9 and “LEfSe” in page 12, the authors should check the manuscript and make sure that there is no obscure abbreviation.

OTU/LEfSe has been added

2. There is no figure 1 in this manuscript and the text jumps into figure 2? (the first mentioned figure throughout this manuscript is in page 9, but the figure number is figure 2-1)

Picture serial number has been corrected

3. Several “LAD SCORE” in page 12 should be “LDA SCORE”.

already edited

4. Figure 2-2 A, B and C, page 12 of the text, the authors show the LDA SCORE of test group on 7th day, LDA SCORE of control group on 21th day and 35th. How about the LDA SCORE of control group on 7th day? And LDA SCORE of test group on 21th day and 35th?

Explanation has been added

5. Figure 2-2, there are annotation like “Control group” and “Trial group” in the figure, will it be better to remove the corresponding Chinese annotation?

Chinese deleted

Reviewer #2: The submission reported the effect of HXBM408 on the bioconversion of DAI and so as the microflora in the rat GI. Following concerns should be addressed before further consideration.

1. Why did the authors choose female Sprague-Dawley rats instead of males? You were only evaluating microflora but not female related cancers?

In this article, the effects of DAI and EQ are estrogen-like effects, which are mainly reflected in female animals.

2. What's the age of your rats?

Rats are 50 days old

3. 3.Please mentioned Pediococcus acidilactici instead of HXBM408 in you content which was misleading.

already edited

4. what's 3500 r/min?（转速） Its not the standard expression.

already edited

5. Why not the authors preform the test in a dose-depentdant manner? How did you confirm the dosage at 1 g/kg?

According to the calculation of the bacterial viability of the bacterial liquid, each rat is fed no less than 10^9^ live bacteria per day

6. What's the difference b/w administration of DAI for bioconversion and direct feeding of Eq?

DAI is the precursor of EQ. The price of EQ is very high. The purpose of this experiment is to make rats have the ability to convert DAI into EQ.

7. From 3.1: The results seemed not really successful, what's the comment from the authors?

After the bacteria liquid stopped gavage, the EQ transformation ability of rats did not increase over time. It may be because Pediococcus acidilactici, as a passing bacteria, failed to permanently colonize the rats, so further research is needed in the later period.

9. Figure 2: Please remove the Chinese words from the content.

Chinese deleted

10. It seems that there was no long-trem effect from the feedding of HXBM408. What's the comment from the authors?

After the bacteria liquid stopped gavage, the EQ transformation ability of rats did not increase over time. It may be because Pediococcus acidilactici, as a passing bacteria, failed to permanently colonize the rats, so further research is needed in the later period.

11. The English style should be revised.

Language has been modified
